# CRISPR-Cas13-Mediated Knockdown of Regulator of G-Protein Signaling 8 (RGS8) Does Not Affect Purkinje Cell Dendritic Development

**DOI:** 10.3389/fcell.2022.854273

**Published:** 2022-05-31

**Authors:** Qin-Wei Wu, Josef P. Kapfhammer

**Affiliations:** Institute of Anatomy, Department of Biomedicine, University of Basel, Basel, Switzerland

**Keywords:** CRISPR, Cas13, RGS8, Purkinje cells, RNA interference, spinocerebellar ataxias, SCA

## Abstract

CRISPR-Cas13 technology is rapidly evolving as it is a very specific tool for RNA editing and interference. Since there are no significant off-target effects via the Cas13-mediated method, it is a promising tool for studying gene function in differentiating neurons. In this study, we designed two crRNA targeting regulator of G-protein signaling 8 (RGS8), which is a signaling molecule associated with spinocerebellar ataxias. Using CRISPR-Cas13 technology, we found that both of crRNAs could specifically achieve RGS8 knockdown. By observing and comparing the dendritic growth of Purkinje cells, we found that CRISPR-Cas13-mediated RGS8 knockdown did not significantly affect Purkinje cell dendritic development. We further tested the role of RGS8 by classical RNAi. Again, the results of the RNAi-mediated RGS8 knockdown showed that reduced RGS8 expression did not significantly affect the dendritic growth of Purkinje cells. This is the first example of CRISPR-Cas13-mediated gene function study in Purkinje cells and establishes CRISPR-Cas13-mediated knockdown as a reliable method for studying gene function in primary neurons.

## Introduction

CRISPR-Cas based methods have greatly simplified RNA-editing and RNA interference studies. For RNA-interference, the novel Class II type VI CRISPR-Cas RNA endonuclease, Cas13, has recently been introduced (Abudayyeh et al., 2017; Cox et al., 2017; Konermann et al., 2018). Cas13 has been identified in bacteria where it serves a component of the anti-phage defense system. Cas13 contains two nucleotide-binding domains (HEPN) associated with RNase activity which are active in higher eukaryotes and prokaryotes. The RNase activity is activated by a crRNA binding to a programmable specific RNA sequence which then forms a Cas13-CRISPR RNA (crRNA) complex, which is capable of destroying a specific RNA due to its strong RNase activity, referred to as cis-RNA cleavage. There may also be non-specific RNA cleavage independent of the binding of the crRNA called trans-RNA cleavage or collateral cleavage. Trans-RNA cleavage can induce collateral effects such as inhibiting cell growth in bacteria, and the collateral effect is used as reporter system in a Cas13-based molecular detection platform for diagnostic purposes (Gootenberg et al., 2017). Collateral cleavage by the activated Cas13-crRNA complexes is typically observed in prokaryotes but was found to be absent in eukaryotic cells. When the Cas13 system was expressed in mammalian cells or plant cells for targeted gene silencing, no collateral activity was detected. Therefore, the main advantage of this technology is a reduced off-targeted effect in the presence of a strong reduction of the target RNA making it potentially superior to classical RNA interference (RNAi) methods (Abudayyeh et al., 2017; Cox et al., 2017). Off-targeted effects caused by classical RNAi mediated gene knockdown complicate the study of gene function during neurodevelopment (Alvarez et al., 2006; Baek et al., 2014), therefore, it is reasonable to consider the advantage of the high specificity of the Cas13-mediated knockdown system. An increasing number of technological variants based on Cas13 have recently been reported for the diagnosis or treatment of RNA viruses including SARS-CoV2 (Abbott et al., 2020; Ackerman et al., 2020; Arizti-Sanz et al., 2020; Fozouni et al., 2021), few studies have focused on gene function in differentiating neuronal cells.

Regulator of G-protein signaling 8 (RGS8) has been reported to be strongly and specifically expressed in cerebellar Purkinje cells (Itoh et al., 2001; Saitoh and Odagiri, 2003). Recently, RGS8 was shown to be associated with the pathology of several forms of spinocerebellar ataxia (SCA), an inherited disease associated with cerebellar degeneration (Gatchel et al., 2008; Dansithong et al., 2015; Wu and Kapfhammer, 2021b). More than 40 types of SCA have been identified and RGS8 was reported to be downregulated in mouse models of SCA1, SCA2, and SCA7 (Gatchel et al., 2008; Dansithong et al., 2015; Klockgether et al., 2019). It is noteworthy that, in a mouse model of SCA14 with a constitutively active PKCγ mutant, RGS8 has been shown to modulate mGluR1-PKCγ signaling in Purkinje cells and overexpression of RGS8 could reduce DHPG induced mGluR1 signaling (Wu and Kapfhammer, 2021b). Considering the transcriptional downregulation of RGS8 in diverse types of SCAs (Gatchel et al., 2008; Dansithong et al., 2015; Wu and Kapfhammer, 2021b), it is desirable to be able to suppress RGS8 expression in order to better understand the role of RGS8. In this study, we designed two crRNAs against RGS8 and achieved a Cas13-based gene knockdown in order to study the effects of RGS8 downregulation in Purkinje cells. By using the Cas13 gene silencing system, we found that knockdown of RGS8 did not significantly affect development of Purkinje cells. Besides, we also used a Purkinje cell specific RNAi approach. Again, downregulation of RGS8 did not affect Purkinje cell dendritic development. This is to our knowledge the first example of a CRISPR-Cas13 mediated gene silencing in Purkinje cells and will help to better understand the role of RGS8 for SCA pathology.

## Materials and Methods

### Animals

All experiments related to this study were done in accordance with the EU Directive 2010/63/EU and have been approved by the veterinary office of the canton of Basel and permitted by Swiss authorities. The mouse line used in this study for primary cerebellar cell cultures was FVB.

### Immunostainings

Immunocytochemistry was performed as described previously (Wu and Kapfhammer, 2020; 2021d; 2021c). Primary dissociated cerebellar cells in culture were fixed in 4% paraformaldehyde at room temperature for 20–30 min. All solution used in this study were made with 100 mM phosphate buffer (PB). Primary antibodies were diluted in a blocking buffer formulated with PB, 3% non-immune goat serum and 0.1–0.3% TritonX-100. Cells were incubated overnight in a cold room at 4°C along with the diluted primary antibodies. The next day, after washing the indicated number of times with PB solution, PB with 0.1% TritonX-100 was used to dilute the corresponding fluorescence-conjugated secondary antibody. The diluted fluorescence-conjugated secondary antibody was then added to the samples and incubated at 4°C overnight or at room temperature for 2 h. Primary antibodies used in this study included: mouse anti-Calbindin D-28K (Swant, Marly, Switzerland), dilution ratio 1:500; chicken anti-GFP (Abcam, Cambridge, United Kingdom), dilution ratio 1:2,000; rabbit anti-GFP (Novus, Zug, Switzerland), dilution ratio 1:2,000; rabbit anti-RGS8 (Invitrogen), dilution ratio 1:500. Secondary antibodies used in this study included: goat anti-rabbit Alexa 488 (Molecular Probes, Invitrogen), dilution ratio 1:500; goat anti-mouse Alexa 568 (Molecular Probes, Invitrogen), dilution ratio 1:500; goat anti-rabbit Alexa 568 (Molecular Probes, Invitrogen), dilution ratio 1:500; goat anti-chicken Alexa 488 (Molecular Probes, Invitrogen), dilution ratio 1:500. The prepared slices were finally mounted using Mowiol (Sigma-Aldrich, Switzerland), air-dried, observed and photographed with an Olympus AX-70 microscope equipped with a Spot Insight digital camera.

### Western Blot

Cells were lysed with RIPA buffer containing phosphatase and protease inhibitors. The protein samples were separated using sodium dodecyl sulphate–polyacrylamide gel electrophoresis (SDS-PAGE) and transferred to a nitrocellulose membrane. TBS containing 5% BSA was prepared as a blocking buffer and the membranes were incubated in the blocking buffer for 1 h at room temperature. After preparing the primary antibody with the blocking buffer, the membrane was incubated in the primary antibody solution overnight. The next day, after washing with TBS-T, the membrane was incubated with blocking buffer containing HRP-labeled or by infrared fluorescent secondary antibodies at room temperature. Protein visualization was performed by ECL (Pierce, Thermo Scientific, Reinach, Switzerland) or by using Odyssey infrared imager with C-Digit software (LI-COR Biosciences, Bad Homburg, Germany) to visualize and quantify protein expression. Primary antibodies used in this study include mouse anti-GAPDH (1:4,000, Proteintech) and mouse anti-FLAG (1:4,000, ORIGENE). Secondary antibodies used in this study include IRDye 800CW Goat anti-Mouse IgG Secondary Antibody (1:10,000, LICOR), IRDye 680LT Goat anti-Rabbit IgG Secondary Antibody (1:10,000, LICOR), anti-mouse HRP conjugate antibody (1:10,000, Promega) and anti-rabbit HRP conjugate antibody (1:10,000, Promega).

### Plasmid Construction

An engineered codon-optimized Cas13 variant from *Leptotrichia wadei* sequences was cloned into a pL7 plasmid in order to achieve cell-specific expression in Purkinje cells. To obtain a high concentration of plasmids for transfection, we purified the expression plasmids using the EndoFree Plasmid Maxi Kit (QIAGEN). The engineered Cas13 sequence was PCR amplified from the pC014-LwCas13a-msfGFP (plasmid #91902, Addgene), and we thank Prof. Feng Zhang for the gift of the plasmid. The target sequence of crRNA RGS8 guide 1 is ATG​AGA​GAG​AAG​CAC​ATC​GAA​AGA​CTC​C and the target sequence of crRNA RGS8 guide 2 is CGG​GTC​TGG​AAA​TCG​ATA​TTC​ACC​TCC​C. The target sequence of RGS8 miR162 is GGA​GTC​TTG​ATG​TGC​TTC​T.

### Cell Cultures and Transfection

The cerebellum of mice on postnatal day 0 was dissected, dissociated, and the cerebellar cells were cultured in glass chambers containing Poly-D-lysine (Wu and Kapfhammer, 2020; 2021d; 2021c). Cells were cultured in the culture medium consisting of 90% Dulbecco’s modified Eagle medium/F-12 nutrient-based medium, 1% glutamax, 1% N2 supplement, and 10% FBS. 500 ul culture medium was added to each well of the glass chambers 2 h after cell transfection. Thereafter, the cell culture medium was changed every 4 days. Cells were kept in culture for 2 weeks prior to fixation. The cell culture medium for this study was obtained from Life Technologies, Zug, Switzerland. According to the instructions for the Neon Transfection System (Thermo Fisher), we set the pulse voltage at 1200 V, pulse width at 30 ms, and pulse number 1 to perform transfection experiments on cerebellar cells, thereby transfecting the indicated plasmids into Purkinje cells.

### Quantitative Analysis

According to the previously reported dendritic quantification method for Purkinje cells we analyzed the Purkinje cell dendritic development in the present study (Wu and Kapfhammer, 2020; 2021d; 2021c). The specific implementation step was to trace the outline of the dendritic tree of Purkinje cells using an image analysis program (ImageJ) to derive the area covered by the dendritic tree. The data were analyzed using GraphPad Prism software (San Diego, United States). The confidence interval was 95%, and *p* < 0.05 was considered as statistically significant. The statistical significance of parameter differences was assessed by a nonparametric two-tailed Mann-Whitney test, one-way ANOVA with multiple comparisons or *t* test.

## Results

### RGS8 Silencing via a CRISPR-Cas13 System in Cerebellar Purkinje Cells

**FIGURE 1:**
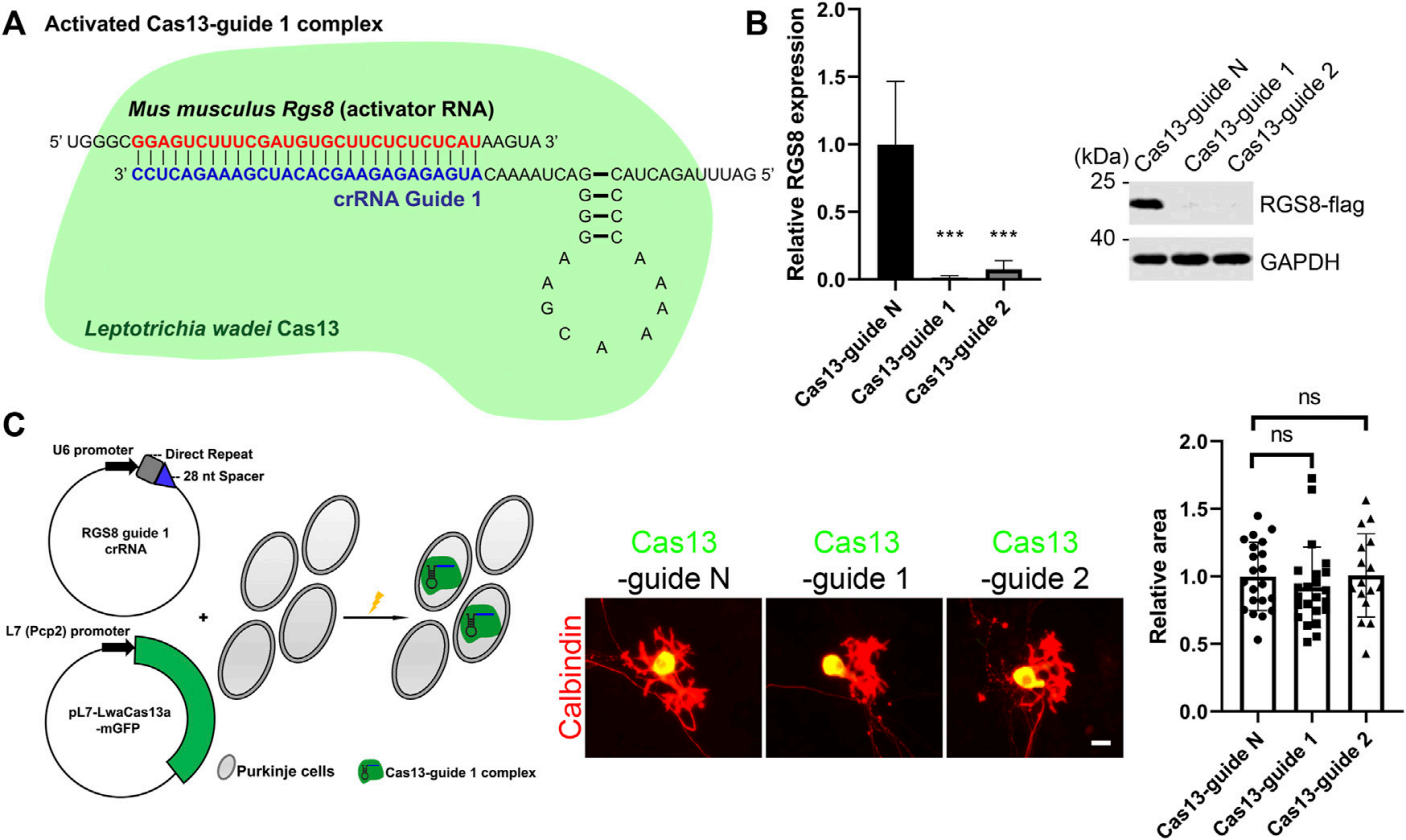
CRISPR-Cas13-mediated RGS8 silencing in cerebellar Purkinje cells. **(A)** This is a schematic diagram of an example of an activated Cas13–guide complex. The target RGS8 RNA acts as an activator RNA. Once the Cas13–guide 1 complex binds to target RGS8 RNA, Cas13–guide 1 complex will be activated and generate RNase activity to cleave the RGS8 transcripts. **(B)** HEK293T cells expressing RGS8-FLAG were transfected with indicated Cas13-guides or control. Western blot of HEK293T cells of indicated Cas13-guides and control group stained for anti-FLAG. Quantification of protein levels from Western blots. The mean value of the control group is 1.00 ± 0.465; the mean value of the is Cas13-guide 1 is 0.01 ± 0.013; the mean value of the is Cas13-guide 2 is 0.07 ± 0.066. n = 4. ***p < 0.001 in one-way ANOVA with multiple comparisons. **(C)** The schematic illustration on the left side of the panel shows that Purkinje cells were transfected Cas13 and crRNA at the setup of the culture. The transfected Purkinje cells were identified by GFP and Calbindin staining in dissociated cultures. The mean values of dendritic area of the Purkinje cells were measured from three independent experiments; controls were the transfected Cas13-guide N (nontargeting) cells. Control: 1.00 ± 0.252, n = 21 cells; Cas13-guide 1: 0.92 ± 0.298, n = 23 cells; Cas13-guide 2 transfection: 1.01 ± 0.309, n = 16 cells. Difference was not statistically significant in one-way ANOVA. Scale bar is 20 µm. Data are expressed as mean ± SD.

In order to study the role of RGS8, we used a Purkinje cell-specific Cas13-mediated system (PCSC13) to achieve knockdown of specific genes as reported recently (Abudayyeh et al., 2017; Wu and Kapfhammer, 2021a). Briefly, this system contained the Purkinje cell-specific L7 (Pcp2) promoter and a mammalian codon-optimized Cas13 variant from *Leptotrichia wadei* with a C-terminal monomeric GFP sequence. In this way, we can identify Purkinje cells expressing Cas13 based on positive GPF expression. Besides, the Cas13 system requires a CRISPR RNA (crRNA) to perform gene knockdown. Normally, the crRNA encodes a 28-nucleotide (nt) spacer. For knockdown of mouse *Rgs8*, we designed two RGS8 guide crRNAs (after that, known as guide 1 and guide 2 respectively). These two crRNA guides are promoted by the U6 promoter and are complementary to the *Rgs8* transcripts. In theory, crRNA (guide 1 for example) firstly binds to Cas13 protein to become a Cas13 guide complex in the cells. Cas13-guide 1 complex is activated by binding to its target mouse *Rgs8* transcript, and it cleaves the *Rgs8* transcript (Figure 1A). We tested the knockdown efficiency of guide 1 and guide 2 via expression of Rgs8-flag in HEK293T cells and observed that both the Cas13-guide 1 and the Cas13-guide 2 complexes could lead to strong gene knockdown for Rgs8-flag expression compared with Cas13-guide N (nontargeting) complex (Figure 1B). Therefore, we co-transfected the crRNAs and pL7-LwaCas13a-mGFP into Purkinje cells on the day we started cerebellar cell culture. After a 2-week culture period, we analyzed the dendritic morphology of Purkinje cells from the control group and the group with activated Cas13-crRNA complex, because Cas13 expression can be identified by the GFP tag (Figure 1C). Since the CRISPR-Cas13 system was expressed mainly in the cell body of Purkinje cells, we are not able to completely label Purkinje cells by anti-GFP staining. For this reason, Purkinje cell dendritic morphology was revealed by anti-Calbindin staining. We compared Purkinje cell dendritic development under the indicated conditions. The mean area of Purkinje cell dendritic outgrowth in group Cas13-guide 1 and guide 2 was not significantly changed compared to the Purkinje cells transfected with Cas13-guide N (nontargeting) complex (Figure 1C). These results demonstrate that the downregulation of Rgs8 is not able to cause abnormality of cerebellar Purkinje cell dendritic growth.

### RGS8 Silencing via a Purkinje Cell Specific miRNA System

**FIGURE 2:**
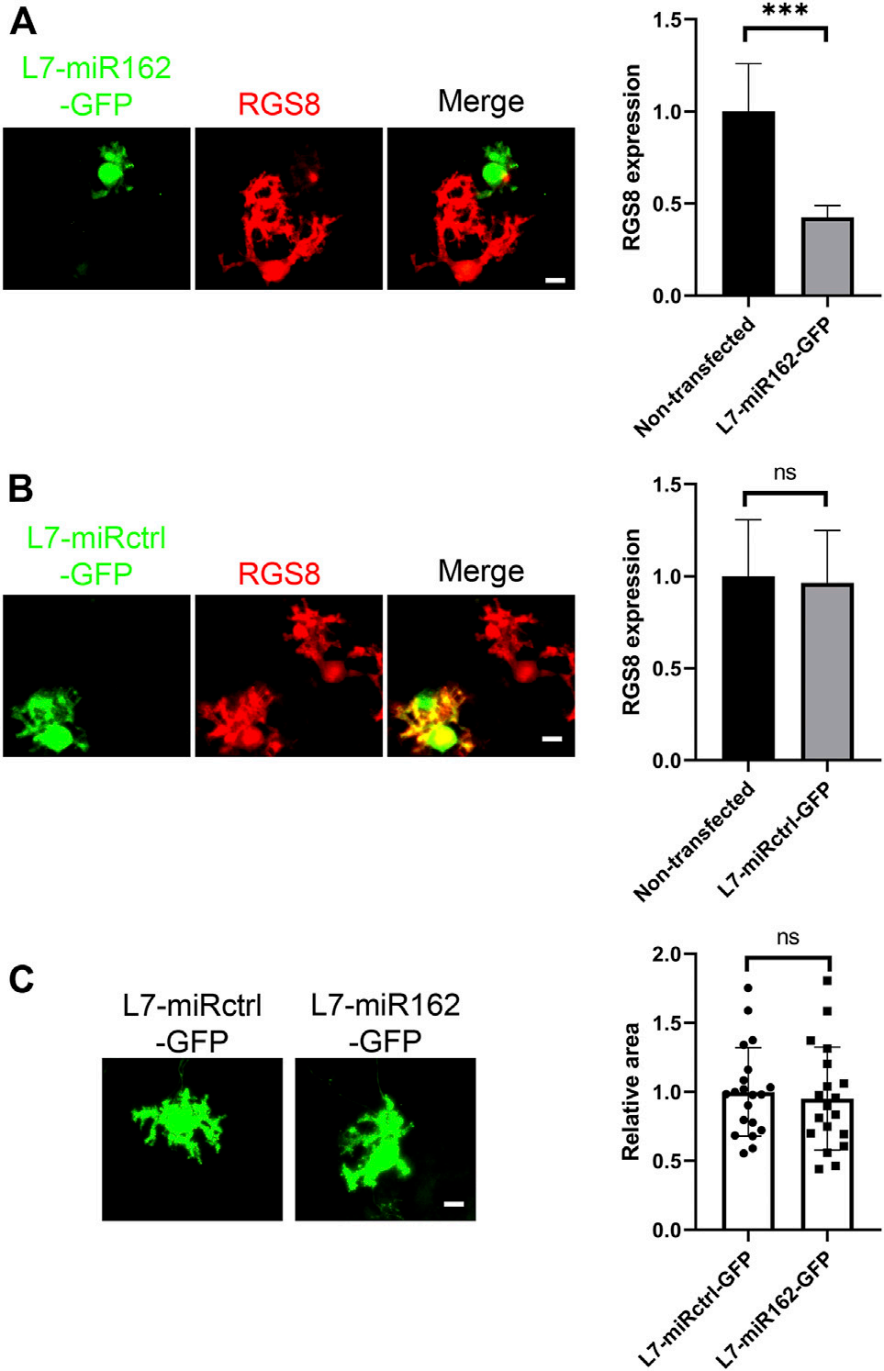
RGS8 silencing by miRNA did not affect Purkinje cell development. **(A)** The transfected Purkinje cells were identified by GFP staining in dissociated cultures and RGS8 protein expression was quantified on GFP-positive Purkinje cells versus neighboring non-transfected Purkinje cells without GFP expression in the culture dish. The mean value of RGS8 expression in L7-miR162-GFP was 0.43 ± 0.064 compared to control cells (1.00 ± 0.260). n = 6, the difference in expression was significant with ***p < 0.001 in *t* test. **(B)** The mean value of RGS8 expression in L7-miRctrl-GFP was 0.96 ± 0.285 compared to control cells (1.00 ± 0.307). n = 6, Difference was not statistically significant in *t* test. Scale bar is 20 µm. Data are expressed as mean ± SD. **(C)** The Purkinje cells transfected with L7-miRctrl-GFP or L7-miR162-GFP were identified by GFP staining in dissociated cultures. The mean values of dendritic area of the Purkinje cells were measured from three independent experiments; L7-miRctrl-GFP: 1.00 ± 0.320, n = 20 cells; L7-miR162-GFP: 0.95 ± 0.373, n = 19 cells; Difference was not statistically significant in the two-tailed Mann-Whitney test. Scale bar is 20 µm. Data are expressed as mean ± SD.

Although PCSC13 worked well and allowed gene knockdown in cerebellar Purkinje cells with considerable level of efficacy and specificity, we had reported in a previous study that the variant of Cas13 used in this system had a neurotoxic action on nerve cell development (Wu and Kapfhammer, 2020; 2021d; 2021c; 2021a). In order to confirm the role of RGS8 downregulation, we also used Purkinje cell specific knockdown based on miRNA. We constructed complementary oligonucleotides specific for the mouse *Rgs8* gene according to the BLOCK-iT RNAi designer method from Life Technologies company. The program generated several RNAi sequences with a predicted rate of knockdown efficiency. We chose the miR-162 sequence and constructed L7-miR162-GFP and L7-miRctrl-GFP (nontargeting) vectors according to the guidelines of generation of Purkinje cell-specific miRNA knockdown constructs. Similar to the Cas13 system visualized by GFP reporter, we identified the expression of miR162 or miRctrl in Purkinje cells based on the expression of the GFP tag. We confirmed that miR162 did achieve an RGS8 knockdown in Purkinje cells (Figure 2A), in contrast to miRctrl (Figure 2B). We also compared the mean area of Purkinje cell dendritic growth from miR162 and miRctrl group cells. Again, the mean area of Purkinje cell dendritic growth in miR162 group was not significantly changed compared to the Purkinje cells transfected with nontargeting miRctrl (Figure 2C). These results demonstrate that the downregulation of RGS8 did not significant affect cerebellar Purkinje cell dendritic development.

## Discussion

The CRISPR-Cas13 method using appropriate guide RNAs can be used to specifically knockdown genes of interest in Purkinje cells and our study demonstrates that the use of CRISPR-Cas13 strategies for gene silencing works very well. An important advantage of the Cas13-based method for the knockdown of transcripts is a much higher specificity compared to RNAi because Cas13 will only be active in the absence of mismatches in the guide-target duplex, having virtually no off-target effects (Abudayyeh et al., 2017). Our findings establish the use of the CRISPR-Cas13 method for RGS8 silencing in postmitotic Purkinje neurons. We found that RGS8 knockdown by either CRISPR-Cas13 or RNAi method did not interfere with Purkinje cell dendritic development.

It is known from previous studies (Abudayyeh et al., 2016, 2017) that Cas13 causes an inhibition of replication and growth in bacteria which was shown to be due to collateral activity. We have shown in our previous work that Cas13 also interferes with developmental processes in neuronal cells such as dendritic outgrowth. This negative effect on neuronal development, however, was shown not to be due to collateral activity of Cas13, but to be associated with a direct toxic effect of Cas13 variants (Wu and Kapfhammer, 2020; 2021d; 2021c; 2021a). Despite this toxic action there is still the advantage of the high specificity of the Cas13 based system for gene silencing. Our findings in this study show that RGS8 knockdown mediated by Cas13 did not cause a significant difference in dendritic outgrowth compared with cells from the control group. Confirming these results, RGS8 knockdown by RNAi did not affect Purkinje cell dendritic development. Taken together our findings suggest that Cas13 mediated gene knockdown is an efficient tool for studies of gene function. Similar to other methods, the obvious advantages come together with negative sides. For example, virus-based gene transduction has become a powerful tool for gene or RNAi delivery *in vitro* and *in vivo*, despite its disadvantage that the viruses infect cells and also express their own genetic material inducing immunogenicity and cytotoxicity (Kim and Eberwine, 2010). Furthermore, virus transduction can cause an inflammatory response and insertional mutations, as the genetic material of the virus integrates randomly into the host genome, which may lead to a disruption of tumor suppressor genes, activation of oncogenes or disruption of other important genes (Woods et al., 2003). RNAi-based non-viral approaches need to introduce foreign reagents by transfection or other delivery methods also known to produce adverse effects. Liposomes, for example, can affect neuronal morphology, growth or viability by lipofection; accelerated gold particles can cause cell damage by the biolistic particle delivery system (Karra and Dahm, 2010). An ideal RNAi method should be able to interfere with the target RNAi with high specificity in order to clarify the loss of function phenotype of the target gene. However, an unspecific knockdown of genes by the use of RNAi, known as off-target effect, has been shown to occur in complex neuronal cell types (Alvarez et al., 2006; Baek et al., 2014). Differential expression analysis showed hundreds of significant off-targets in the RNAi conditions but none in CRISPR-Cas13 knockdown conditions (Abudayyeh et al., 2017), suggesting that CRISPR-Cas13 will be a suitable tool for studying gene function in complex neuronal cell types. Low cytotoxicity but high specificity with an acceptable adverse effect on dendritic growth might make it still a superior alternative for gene function studies. Our study provides a first example for conditional gene silencing using CRISPR-Cas13 to investigate gene function in Purkinje cells.

Recent studies have implicated RGS8 as a molecule which is dysregulated in several forms of SCA. It has been suggested that the reduction of RGS8 expression found in SCA2 contributes to an overstimulation of the mGluR1-Gαq pathway that contributes to pathology in SCA2 (Dansithong et al., 2015). In our previous work we have shown that overexpression of RGS8 can protect Purkinje cell dendritic trees from the negative effects of pharmacological mGluR1 activation, supporting the concept that RGS8 is down regulating the activity of the Gαq subunit to inhibit mGluR1 signaling (Wu and Kapfhammer, 2021b). Interestingly, loss of RGS8 in an RGS8 null mutant mouse model goes together with normal development of Purkinje cells and the mice show no signs of ataxia (Kuwata et al., 2007). These results agree with our finding from this study showing that acute knockdown of RGS8 by two different technologies (CRISPR-Cas13 or miRNA) did not cause a significant inhibition of Purkinje cell dendritic development. These data clearly indicate that RGS8 downregulation by itself may not be a problem for Purkinje cells. Only when it goes together with increased signaling of the mGluR1-Gαq pathway it appears to become problematic, because under these conditions it may have an important protective function which in its absence cannot be recruited (Dansithong et al., 2015; Wu and Kapfhammer, 2021b). Taken together, all these studies are best compatible with a role of RGS8 as a protective modifier for increased mGluR1 signaling in Purkinje cells. Many RGS family members are expressed in brain and may play similar modifier roles. The loss of function caused by deficient RGS8 might be compensated for by its family members (Squires et al., 2018). Such a compensation may also occur in the testis in which RGS8 is also strongly expressed, but RGS8 knock-out mice are fertile (Saitoh et al., 1999; Kuwata et al., 2007). RGS22, which is shown to interact with Gαq/11, is specifically expressed in mouse testis (Hu et al., 2008) and may be a candidate RGS member which could compensate for the loss of RGS8 function in testis. RGS4, which shows expression in brain and cerebellum, is phylogenetically close to RGS8 and belongs to the R4 sub-family. The highest level of RGS4 mRNA expression is in the Purkinje cell layer with relatively modest expression in the granule cell layer (Ingi and Aoki, 2002; Squires et al., 2018). RGS4 can inhibit mGluR1 and mGluR5 coupling to Gq pathways in some brain regions (Saugstad et al., 1998), indicating that RGS4 is a potential candidate which might be compensating for the loss of RGS8 function in Purkinje cells. To further clarify and define the role of RGS8 for the pathology of SCAs it needs to be achieved by further studies.

## Data Availability

The original contributions presented in the study are included in the article/supplementary material, and further inquiries can be directed to the corresponding author.
